# Ion Capture and Release Ability of Glass Ionomer Cement Containing Nanoporous Silica Particles with Different Pore and Particle Size

**DOI:** 10.3390/ma14195742

**Published:** 2021-10-01

**Authors:** Ryoshun Endo, Ko Nakanishi, Yosuke Bando, Shigeaki Abe, Haruhi Maruoka, Mariko Nakamura, Tsukasa Akasaka, Yasuhiro Yoshida, Yoshiaki Sato

**Affiliations:** 1 Department of Orthodontics, Faculty of Dental Medicine, Hokkaido University, Kita13, Nishi7, Kita-ku, Sapporo 060-8586, Japan; e.ryoshun@den.hokudai.ac.jp (R.E.); y.bandou24@den.hokudai.ac.jp (Y.B.); h.maruoka@den.hokudai.ac.jp (H.M.); yoshi-ma@den.hokudai.ac.jp (Y.S.); 2 Department of Biomaterials and Bioengineering, Faculty of Dental Medicine, Hokkaido University, Kita13, Nishi7, Kita-ku, Sapporo 060-8586, Japan; akasaka@den.hokudai.ac.jp (T.A.); yasuhiro@den.hokudai.ac.jp (Y.Y.); 3 Department of Dental and Biomedical Materials Science, Graduate School of Biomedical Sciences, Nagasaki University, Sakamoto 1-7-1, Nagasaki 852-8102, Japan; sabe_den@nagasaki-u.ac.jp; 4 School of Clinical Psychology, Kyushu University of Health and Welfare, yoshinocho 1714-1, Nobeoka 882-8508, Japan; marin@phoenix.ac.jp

**Keywords:** nanoporous silica, glass ionomer cement, ion capture/release abilities, pore size, particle sizes

## Abstract

This study prepared glass ionomer cement (GIC) containing nanoporous silica (NPS) (GIC–NPS) at 5 wt% concentrations using 3 types of NPS with different pore and particle sizes and evaluated the differences in their cationic ion capture/release abilities and mechanical properties. The cationic water-soluble dye was used as cationic ion. The test GIC–NPS complexes captured dyes by immersion in 1 wt% dye solutions. All the GIC–NPS complexes released dyes for 28 d, and the amount of dye released from the complexes increased with decreasing pore size; however, the particle size of NPS did not affect the amount of dye released. Additionally, GIC–NPS was able to recharge the dye, and the amount of released the dye by the complexes after recharge was almost identical to the amount released on the first charge. Although not significantly different, the compressive strength of GIC–NPS was slightly greater than that of GIC without NPS regardless of the type of NPS. These results suggest that the degree of capture and release of cationic molecules, such as drugs, can be controlled by optimizing the pore size of NPS without sacrificing its mechanical strength when its content is 5 wt%.

## 1. Introduction

During orthodontic treatment, maintaining proper oral hygiene around fixed appliances with brackets or bands is difficult and presents an increased risk of caries around these fixed appliances. Caries is often found after debonding fixed appliances. In fact, there are many reports on the relationship between caries and fixed appliances. Richter et al. [1] reported that the incidence of patients who developed at least one new white spot lesion during orthodontic treatment using fixed appliances was reported to be 72.9%, and 2.3% of these patients had cavitated lesions. Furthermore, Gorelick et al. [2] reported that 10.8% of bonded teeth and 12.0% of banded teeth have white spot lesions after orthodontic treatment. To solve this problem, we sought to develop a novel dental adhesive that could gradually release a drug preventing caries during orthodontic treatment.

Recently, nanomaterials have gained much attention in various fields with advancements in nanotechnology. In dentistry, nanomaterials have been widely studied for their use in improving dental fillings, hypersensitivity agents, dental implants, and others. For example, carbon nanotubes have been used in dental fillings and various other applications because of their unique mechanical and electrical properties [3]. Hydroxy apatite nanoparticles have been used for treating dental hypersensitivity [4] and retarding the demineralization of enamel [5] due to binding to dentin apatite and tooth enamel. Silica nanoparticles have been used as dental fillers [6,7] and as an abrasive to polish teeth [8]. Among silica nanoparticles, nanoporous silica (NPS) has been investigated extensively over the past two decades because of its desirable structural properties, such as its large surface area, tunable pore sizes, and reactive surfaces [9,10,11,12]. NPS can function as a drug carrier as it can capture and release molecules. It has been reported that NPS can capture and release fenbufen [13], silver [14], and chlorhexidine [15,16]. Despite NPS’s accessible drug carrier properties, there are few reports of the application of NPS in dentistry [16,17,18]. We therefore focused on using NPS as a functional drug carrier. In a previous study [19], we reported that NPS added into glass ionomer cements (GIC), which is one of the most used dental cements, captured cationic dye and slowly released it for 2 weeks. In addition, it was found that NPS could recharge the dye after releasing it. However, this previous study did not determine the effect of NPS pore and particle size on its ability to capture and release molecules. There have been few reports of NPS added into GIC for a drug carrier release drug, and molecular and recharge ability and effect of pore and particle size were not evaluated [17,18].

The aim of this study was to prepare GIC containing NPS (GIC–NPS) using NPS with different pore and particle sizes and to evaluate the different GIC–NPS complexes’ cationic ion capture and release abilities, recharge ability, and mechanical properties.

## 2. Materials and Methods

### 2.1. Materials

Three types of NPS, 3.0 μm and 4.0 nm (NPS3-4), 3.0 μm and 2.0 nm (NPS3-2), and 0.5 μm and 2.0 nm (NPS0.5-2) particle and pore size, respectively, were purchased from Sigma-Aldrich. The three types of NPS profiles are summarized in Table 1. Glass ionomer cement (Fuji I, GC, Tokyo, Japan) was used and rhodamine B (RhB) (Sigma-Aldrich, St. Louis, MO, USA) was selected as the cationic water-soluble dye.

### 2.2. Preparation of GIC-NPS Test Pieces and Controls

NPS3-4, NPS3-2, and NPS0.5-2 were mixed into the powder component of GIC at 5 wt% concentrations. To evaluate their dye capture and release abilities as well as their mechanical properties, the test pieces (GIC–NPS3-4, GIC–NPS3-2, GIC–NPS0.5-2) were first molded by mixing the obtained powder and liquid of GIC as specified by the manufacturer [20]. The test pieces were 1 mm thick and 10 mm in diameter to evaluate dye capture and release ability and 10 mm high and 6 mm in diameter to evaluate mechanical properties. Pure GIC was molded into the same sizes to be used as experimental controls.

### 2.3. Evaluation of Dye Capture and Release Abilities

For dye capture, the test pellets of GIC–NPS3-4, GIC–NPS3-2, and GIC–NPS0.5-2 were immersed in 1 wt% RhB aqueous solutions at 37 °C for 24 h. After 24 h, the pellets were removed from the RhB aqueous solutions and washed with distilled water. The obtained test pellets were immersed in 5 mL distilled water at 37 °C for 24 h. The pellets were then removed from the solution and re-immersed in fresh distilled water. The distilled water was changed every day for 28 d. The absorption spectra of the obtained supernatants were measured using a UV–visible spectrophotometer (V-650, Jasco, Tokyo, Japan) to evaluate the amount of dye released each day. After 28 d, to evaluate recharge ability, the pellets of GIC–NPS3-2 and GIC–NPS3-4 were again immersed in 1 wt% RhB aqueous solution at 37 °C for 24 h and amount of released dye was measured in the same way. The GIC controls were also treated and measured in the same manner as the test pellets.

### 2.4. Evaluation of Mechanical Property

The compressive strength of the GIC–NPS3-4, GIC–NPS3-2, and GIC–NPS0.5-2 complexes were measured using a universal testing machine (Model3366, Instron, Canton, OH, USA) for the evaluation of mechanical properties. Five samples were tested, respectively. The cross-head speed was set at 1 mm/min. The GIC controls were also measured in the same manner. The compressive strength was calculated by the following formula:

(1) $$\sigma =\frac{F}{A}$$

where σ is the compressive strength, *F* is the compressive force, and *A* is the cross-section area. The results were statistically analyzed using a Steel–Dwass test.

## 3. Results

### 3.1. Dye Capture and Release Properties

**Figure 1:**
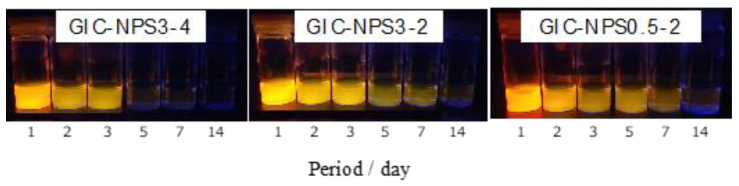
Photo images under ultraviolet light of each supernatant in which GIC–NPS3-4, GIC–NPS3-2, or GIC–NPS0.5-2 were immersed for different periods.

Images taken under ultraviolet light of each supernatant in which GIC–NPS3-4, GIC–NPS3-2, or GIC–NPS0.5-2 were immersed for the same time periods are shown in Figure 1. The color of the supernatant for all three complexes gradually became fainter as the immersion time increased. The supernatant GIC–NPS3-2 and GIC–NPS0.5-2 were approximately the same color despite immersion times. The color of the supernatant of GIC–NPS3-4 was clearly fainter at day 5, 7, and 14 than those of GIC–NPS3-2 and GIC–NPS0.5-2.

**Figure 2:**
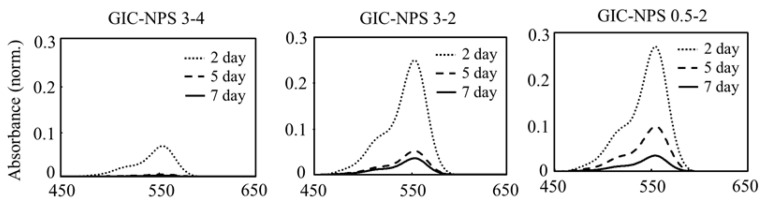
The absorption spectra of the supernatants in which GIC–NPS3-4, GIC–NPS3-2, or GIC–NPS0.5-2 were immersed for 2, 5, and 7 days.

Figure 2 shows the absorption spectra of the supernatants in which GIC-NPS3-4, GIC-NPS3-2, or GIC-NPS0.5-2 were immersed for 2, 5, and 7 days. The supernatants’ absorption spectra exhibited peaks at 554 nm corresponding to RhB [21]. Regardless of the pore and particle size of NPS, the absorption intensities gradually decreased depending on the time period of immersion. The intensity of GIC–NPS3-4 was less than that of GIC–NPS3-2 and GIC–NPS0.5-2.

**Figure 3:**
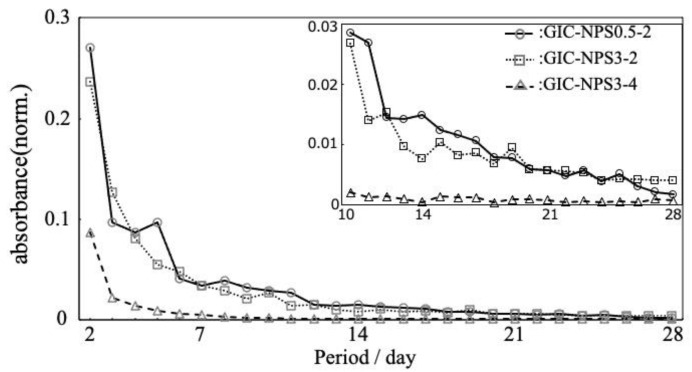
The time profile of the absorbance of released RhB from GIC–NPS.

The time profile of the absorbance of released RhB from GIC–NPS is depicted in Figure 3. Though the eluted RhB was detected from all the GIC–NPS pieces for 28 days, the amount of released RhB gradually decreased depending on the immersion time. GIC–NPS3-2 and GIC–NPS0.5-2 both displayed very similar time profiles of RhB release. In contrast, the amount of released dye from GIC–NPS3-4 was less than that of GIC–NPS3-2 and GIC–NPS0.5-2.

### 3.2. Recharge Ability of GIC–NPS

**Figure 4:**
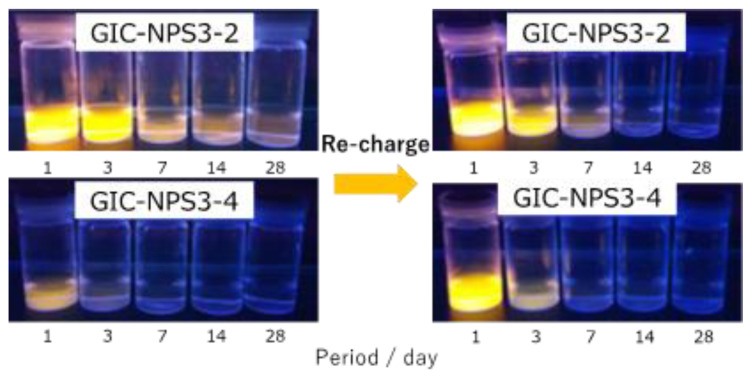
Color change of GIC-NPS3-2 and GIC-NPS3-4 supernatant under ultraviolet light during initial charge and recharge.

The supernatants recovered from both GIC–NPS3-4 and GIC–NPS3-2 immersed for different time periods after the dye recharge process are displayed in Figure 4. Compared to Figure 1, the color of the supernatants after recharge were lighter than those after their first charge. Similar to the results seen after the first charge, the color of GIC–NPS3-2′s supernatant was darker than that of GIC–NPS3-4′s.

**Figure 5:**
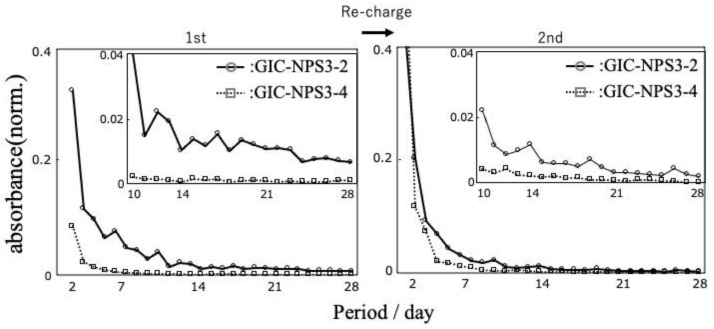
The time profile comparison of the absorbance of released RhB from GIC–NPS first charge and recharge.

The time profiles of the absorbance of released RhB from GIC–NPS after recharge reveals the tendency of GIC–NPS to release RhB on recharge to be similar, albeit slightly reduced, to its release on the first charge (Figure 5).

### 3.3. Mechanical Property of GIC–NPS

**Figure 6:**
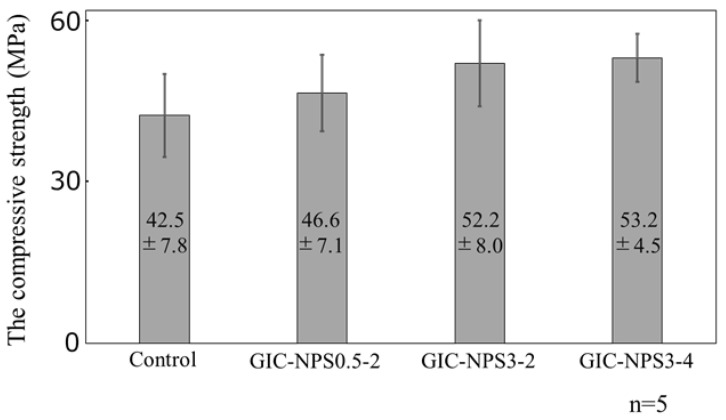
The compressive strength of GIC–NPS.

Figure 6 shows the compressive strength of GIC–NPS3-4, GIC–NPS3-2, and GIC–NPS0.5-2. Although the compressive strength of GIC containing NPS in 5 wt% concentration was slightly higher than that of the control (i.e., GIC without NPS) regardless of the type of NPS, there were no significant differences between the GIC–NPS complexes. 

## 4. Discussion

### 4.1. Materials

GIC is commonly used in both orthodontic and dental treatments. A major reason for its popularity is because GIC has demonstrated a sustained-release property of fluorine. Fluorine ions are well known to protect teeth from becoming dental caries due to fluorapatite formation [22,23], remineralization [22,24], and antienzymatic action [22,25]. With its sustained release properties of fluorine, GIC is better at preventing caries than other dental adhesives. The occurrence of dental caries in the interfaces between the dental adhesives and the teeth is not entirely preventable. By imbuing GIC with drug- or other ion-releasing properties, it can more efficiently inhibit the formation of caries at the adhesive interface; thus, we focused on GIC as the matrix in this study. 

The RhB used in this study has a positive charge and is water soluble. In addition, RhB is sensitive to both optical and spectroscopic analyses. Thus, RhB’s properties make it suitable for the elucidation of the cationic drug capture and release properties of GIC–NPS pieces. As a result, we succeeded in confirming the effects of NPS’s pore and particle size on the pieces’ dye capture and release abilities visually (Figure 1).

### 4.2. Dye Capture and Release Properties

Silica’s surface has a negative charge [26] and therefore its negative surface attracts positively-charged RhB. In addition, because RhB is 18.54 Å × 14.35 Å × 9.14 Å [27] in size, it can penetrate the pores of NPS, with pore sizes of 2 or 4 nm. Therefore, it was hypothesized that GIC–NPS’s dye capture and release properties were dependent on NPS’s total surface area including surface area of its pores. According to the manufacturer, the surface area of both NPS3-2 and NPS0.5-2 is 900–1100 m^2^/g and the surface area of NPS3-4 is 300–400 m^2^/g. Since the surface area of NPS3-2 and NPS0.5-2 are equal, we found that both complexes’ dye capture and release abilities were the same regardless of NPS’s particle size. Furthermore, because the surface area of NPS3-2 was greater than that of NPS3-4, the amount of dye released from the GIC–NPS complex using NPS with a pore size of 2.0 nm was more than the complex using NPS with a pore size of 4.0 nm (Figure 3). When the pore size of NPS is large enough to allow penetration by a drug molecule, the amount of drug captured/released from NPS can be controlled by optimizing its surface area and pore size without having to vary its concentration in GIC. In order to consider the effect of surface area of NPS in more detail, it is necessary to measure the specific surface area using BET or t-plot methods in future work.

In this study, all the GIC–NPS complexes gradually released a model drug (RhB) over the 28 d time period. In the case of GIC–NPS3-4 and GIC–NPS3-2, after the dye recharge process, both again exhibited RhB-release behavior for 28 d. If the performance of the GIC-NPS can be reproduced with antimicrobial substances, the following applications can be considered in actual clinical practice. In general, patients return to the hospital or office every 4 weeks during the course of dynamic orthodontic treatment. During the course of treatment, patients can reduce the risk of caries by taking advantage of the drug-release behavior of GIC–NPS. In addition, GIC–NPS’s effective drug-release period can be increased when the drug is recharged during the patients’ hospital or office visits. This results in GIC–NPS’s continuous effectiveness in reducing the risk of caries during the course of orthodontic treatment. Additionally, it may become possible for patients to recharge the drug themselves at home if recharging drug methods become easier. Therefore, the effect of the drug can be maintained even for patients who do not visit the hospital for a longer period whether for orthodontic treatment or other treatment. The importance of developing easy and manageable drug recharging methods needs to be considered in future work.

### 4.3. Mechanical Properties

There are many reports regarding the mechanical properties of GIC with the addition of particles [17,28,29,30]. Alatawi et al. added hydroxyapatite nanoparticles to GIC at concentrations up to 10 wt% and they found that the GIC containing hydroxyapatite nanoparticles had a greater compressive strength than GIC alone at all concentrations [28]. Elsaka et al. reported that the compressive strength of GIC containing TiO_2_ nanoparticles increased in concentrations up to 5 wt% and decreased at 7 wt% compared to GIC alone [29]. In this study, there were no significant differences between the various GIC–NPS complexes although the complexes did exhibit a slightly greater compressive strength than the GIC controls. It is known that the GIC contains glass particles as filers and the size of glass particles is adjusted to 1.5–40 μm. It is thought that the compressive strength of GIC–NPS is improved when the empty spaces between the large GIC glass particles are filled with NPS. However, NPS may interfere with GIC’s normal curing reaction when the concentration of NPS added to GIC increases [17]. Therefore, at a certain point, increasing the amount of NPS added to GIC may reduce the compressive strength of the GIC–NPS complex.

Incidentally, the effect of different pore and particle sizes of NPS on the compressive strength of the complexes was not confirmed in this study. When Alobiedy et al. added ZrO_2_ with particle sizes of 65 μm or 20 nm to GIC and evaluated the compressive strength of GIC containing ZrO_2_, the large ZrO_2_ particle improved the compressive strength of GIC more than small ZrO_2_ particle [30]. In this study, the compressive strength of GIC–NPS3-2 and GIC–NPS0.5-2 was considered almost equal because the difference in NPS’s particle size is small.

One of the factors affecting the mechanical properties is the dispersion of filler particles in matrix. It is reported that the smaller filler particles disperse well in matrix [31,32,33]. However, the strength of composites appears to be more efficiently enhanced when filler particles arrange in complex structured aggregates than when they are perfectly dispersed [34]. The mechanisms of reinforcement for adding the filler still leave unknown parts, such as the interaction between the filler and the polymer [35]. This is an area that needs to be clarified in order to accurately control the physical properties of composites in the future.

## 5. Conclusions

In this study, we prepared GIC-containing NPS using NPS with three different pore and particle sizes (3.0 μm and 4.0 nm, 3.0 μm and 2.0 nm, 0.5 μm and 2.0 nm, pore and particle size, respectively), and investigated the effects of particle and pore size on the complexes’ cationic ion capture and release abilities and mechanical properties. All the GIC–NPS complexes released cationic ions for 28 d, and GIC–NPS3-2 and GIC–NPS0.5-2 released more cationic ions than GIC–NPS3-4. Interestingly, the amount of cationic ion released from GIC–NPS3-2 and GIC–NPS0.5-2 were almost identical despite their different particle sizes. Based on GIC’s mechanical properties, the compressive strength of GIC containing NPS at the concentration of 5 wt% was slightly greater than that of the GIC control, although the strength was basically independent of the particle and pore size of NPS. These results suggest that GIC–NPS’s degree of cationic drug capture and release can be controlled by optimizing the pore size of NPS within the piece without sacrificing its mechanical strength when the NPS content is 5 wt%.

## Figures and Tables

**Table 1:** The three types of NPS profiles.

	Diameter /mm	Pore Size /mm	Surface Area /m^2^·g^−2^
NPS3-4	3	4	300–400
NPS3-2	3	2	900–1000
NPS0.5-2	0.5	2	900–1000
